# Whole-Genome Comparison Reveals Structural Variations behind Heading Leaf Trait in *Brassica oleracea*

**DOI:** 10.3390/ijms24044063

**Published:** 2023-02-17

**Authors:** Gaoxiang Ji, Ying Long, Guangqin Cai, Guixin Yan, Jinfeng Wu, Fugui Zhang, Lixia Li, Hao Li, Qian Huang, Jinxiong Shen, Xiaoming Wu

**Affiliations:** 1 Key Laboratory of Biology and Genetic Improvement of Oil Crops, Ministry of Agriculture and Rural Affairs, Oil Crops Research Institute of the Chinese Academy of Agricultural Sciences, Wuhan 430062, China; 2 School of Life and Health Sciences, Hunan University of Science and Technology, Xiangtan 411201, China; 3 National Key Laboratory of Crop Genetic Improvement, Huazhong Agricultural University, Wuhan 430072, China

**Keywords:** *Brassica oleracea*, heading trait, comparative genomic, structure variations

## Abstract

*Brassica oleracea* displays remarkable morphological variations. It intrigued researchers to study the underlying cause of the enormous diversification of this organism. However, genomic variations in complex heading traits are less known in *B. oleracea*. Herein, we performed a comparative population genomics analysis to explore structural variations (SVs) responsible for heading trait formation in *B. oleracea*. Synteny analysis showed that chromosomes C1 and C2 of *B. oleracea* (CC) shared strong collinearity with A01 and A02 of *B. rapa* (AA), respectively. Two historical events, whole genome triplication (WGT) of *Brassica* species and differentiation time between AA and CC genomes, were observed clearly by phylogenetic and Ks analysis. By comparing heading and non-heading populations of *B. oleracea* genomes, we found extensive SVs during the diversification of the *B. oleracea* genome. We identified 1205 SVs that have an impact on 545 genes and might be associated with the heading trait of cabbage. Overlapping the genes affected by SVs and the differentially expressed genes identified by RNA-seq analysis, we identified six vital candidate genes that may be related to heading trait formation in cabbage. Further, qRT-PCR experiments also verified that six genes were differentially expressed between heading leaves and non-heading leaves, respectively. Collectively, we used available genomes to conduct a comparison population genome analysis and identify candidate genes for the heading trait of cabbage, which provides insight into the underlying reason for heading trait formation in *B. oleracea*.

## 1. Introduction

*Brassica oleracea* (CC, 2n = 2x = 18) is one of the most important vegetable species for human diets, which includes cabbage (var. *capitata*), cauliflower (var. *botrytis*), broccoli (var. *italica*), Brussels sprout (var. *gemmifera*), kohlrabi (var. *gongylodes*), kale (var. *acephala*), and Chinese kale (var. *alboglabra*). They are rich in carotenoids and glucosinolates (GSLs), functioning as a plant defense against pathogens, and have anticancer properties for human health [1]. In 2019 and 2020, the global market production of *B. oleracea* vegetables was approximately 70.2 and 70.8 million tons, with values of 18.96 and 18.18 billion dollars, respectively (data from FAO: http://faostat.fao.org/, accessed on 7 July 2022).

Cabbage is one of the most widely grown crops of *B. oleracea* and provides edible leaf organs for human consumption [2]. The heading trait of cabbage is an important agronomic trait that affects the yield and quality of cabbage [3]. It is also a typical domesticated trait that can be used for identifying signals of the artificial selection of cabbage [4]. Recently, some quantitative trait loci (QTLs) [3,5] and three candidate genes, *BoKAN2.2*, *BoBRX.2*, and *BoATHB15.2* [4], associated with this complex heading trait have been identified. However, there are no studies of heading trait morphology using structural variations (SVs) in cabbage.

Within the last two decades, the advent of high-throughput sequencing technologies has offered an excellent opportunity for exploring genomics and population genomics in many important crops, such as rice [6,7,8], maize [9], cotton [10,11], and rapeseed [12,13,14]. For *Brassica oleracea* plants, a draft genome of *B. oleracea* named line02-12 (a cabbage type) was first released in 2014 [1]. The genome of To1000, belonging to the Chinese kale type, was decoded in the same year [15]. A chromosome-scale broccoli genome named HDEM was encoded using Oxford Nanopore Technology (ONT) in 2018 [16]. Then, a high-quality reference genome for cabbage line D134 was obtained with the single-molecule real-time (SMRT) sequencing technique in 2020 [17]. An improved version of line02-12 was declared in the same year on the basis of the SMART sequencing technique [18]. Two *B. oleracea* genomes, cabbage type OX-heart and cauliflower type Korso, were reported using the SMART technique in recent research [19]. Based on these reference genomes, population genomics research was performed to study the origin, domestication, and morphotype diversification of *B. oleracea* [4,20,21]. Although six genomes of *B. oleracea* are available, comprehensive comparative genome analysis has rarely been reported. 

Here, we used six high-contiguity *B. oleracea* genomes to conduct a comparative genome analysis. Through synteny analysis, we found that chromosomes C1 and C2 in *B. oleracea* share stronger collinearity with A01 and A02 in *B. rapa* than other chromosomes. Based on 10,950 single-copy ortholog genes, we constructed a phylogenetic tree. Two historical events were observed clearly through synonymous substitution rate (Ks) analysis. In addition, we identified extensive sequence variations containing single nucleotide polymorphisms (SNPs) and small insertions and deletions (InDels) and structural variations (SVs) among these six *B. oleracea* genomes. In particular, we identified six candidate genes affected by SVs that may be involved in heading trait formation in cabbage. In conclusion, we carried out an overall comparative population genomic analysis of six available high-quality *B. oleracea* genomes and 280 *B. oleracea* genome datasets. The results revealed the genetic variations and phylogenetic relationships within the *B. oleracea* genome. Moreover, this study provides useful information for further accelerating the molecular breeding of *B. oleracea* crops.

## 2. Results

### 2.1. Comparison of Genome Features among Six B. oleracea Genomes

**Figure 1:**
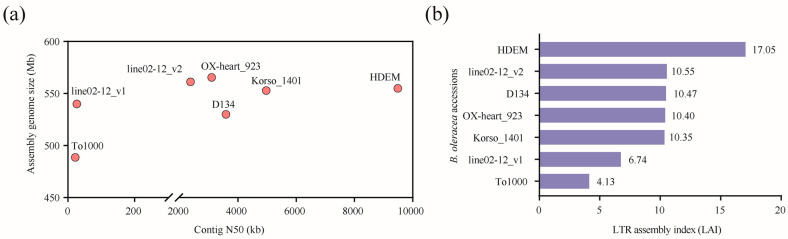
Comparison of assembly genome sizes, contig N50 (**a**), and LAI (**b**) of six *Brassica oleracea* genomes.

The estimated genome size of *B. oleracea* ranges from 567 to 660 Mb. The assembled genome size represented more than 75% of the estimated size of the six genomes (Table S1). The contig N50 of HDEM, OX-heart, Korso, line02-12_v2, and D134, which were assembled by the ONT and/or SMART technologies, is longer than that in To1000 and line02-12_v1 assembled by short reads (Figure 1a). In addition, we found the largest assembly genome size and the greatest number of protein-coding genes annotated in the OX-heart genome (Figure 1a; Table S1). To further assess the quality of the six genomes, we calculated their long terminal repeat (LTR) assembly index (LAI) scores. As a result, the HDEM genome has the highest LAI of 17.05. line02-12_v2, D134, OX-heart, and Korso have LAI values of 10.55, 10.47, 10.40, and 10.35, respectively. These five genomes are categorized at the reference genome level (10 ≤ LAI < 20). However, line02-12_v1 and To1000 are categorized at the draft genome level (0 ≤ LAI < 10) (Figure 1b). These two genomes were assembled mainly by short reads. Therefore, we selected HDEM as the reference genome for further study. 

### 2.2. Gene Synteny and Phylogenetic Analysis of B. oleracea

**Figure 2:**
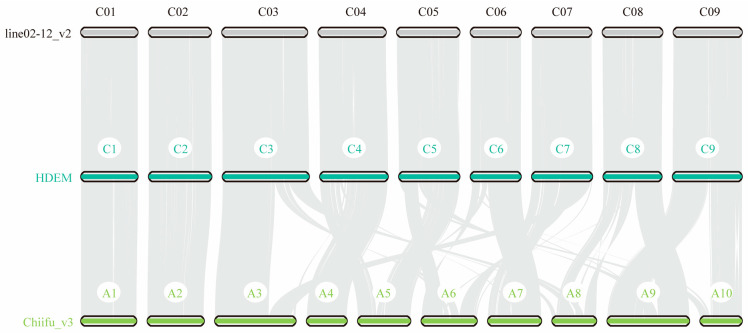
Chromosome-level genome alignment of the *B. oleracea* line02-12_v2 genome (**top**) and *B. rapa* Chiifu_v3 genome (**bottom**) against the HDEM genome (**center**).

We collected six available *B. oleracea* accessions containing HDEM, OX-heart, Korso, line02-12_v2, D134, and To1000 (Table S1) and one *B. rapa* accession, Chiifu_v3, to detect syntenic blocks among seven genomes. As a consequence, there were 32,074–38,418 collinear gene pairs among the six *B. oleracea* genomes (CC) (Figure S1a–e). Meanwhile, we identified 29,066 collinear gene pairs in *B. rapa* Chiifu_v3 (AA) compared with *B. oleracea* HDEM (CC) (Figure S1f). These results suggest that there are more collinear gene pairs in intraspecies than in interspecies. Interestingly, we found that chromosomes C1 and C2 in *B. oleracea* (CC) share stronger collinearity with A01 and A02 in *B. rapa* (AA), respectively, than other chromosomes (Figure 2). It was also found between the AA and CC subgenomes in the allotetraploid *B. napus* (AACC) species [22]. 

**Figure 3:**
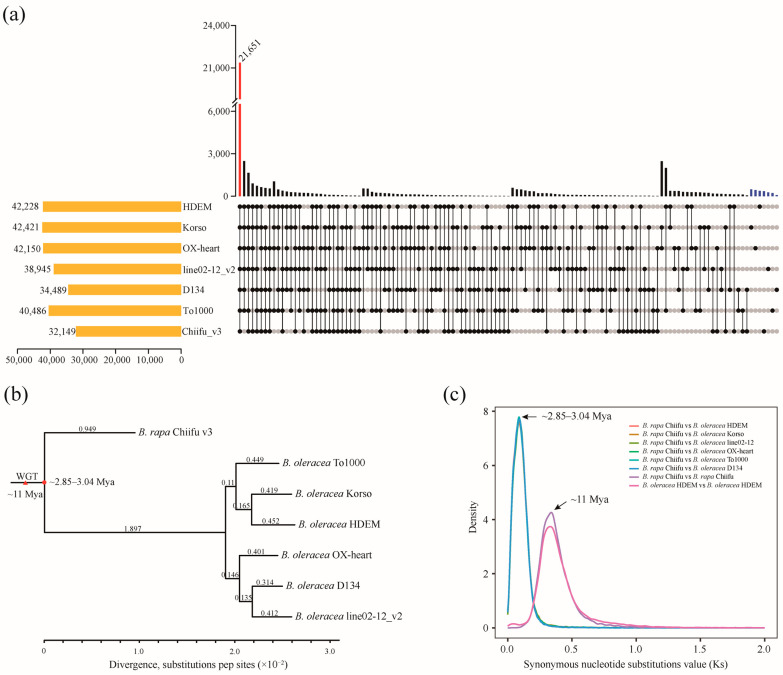
Evolutionary analysis of *B. oleracea*. (**a**) An upset diagram shows unique and shared gene families between six *B. oleracea* and *B. rapa* Chiifu genomes. The number, along with the histograms, show the clusters in each accession. (**b**) Single-copy gene-based phylogenetic relationship for 6 *B. oleracea* accessions with Chiifu as an outgroup. A whole-genome triplication (WGT) event of *Brassica* species is noted by a red triangle. The point of the differentiation time of *B. rapa* (AA) and *B. oleracea* (CC) is marked by a red solid circle (Mya, million years ago). (**c**) Density plots of synonymous substitutions (Ks) of Chiifu and other plant species are represented by columns of different colors. The arrows marked at the peak of Ks, from left to right, represent the differentiation time of *B. rapa* (AA) and *B. oleracea* (CC) and the time of *Brassica*-specific WGT event, respectively.

To evaluate the phylogenetic relationship of *B. oleracea*, we utilized the protein sequences of six *B. oleracea* genomes, including HDEM, OX-heart, Korso, line02-12_v2, D13, and To1000, as well as the *B. rapa* Chiifu_v3 genome (Figure 3a) to identify orthologous gene groups by OrthoFinder [23] (see Methods for details). A total of 388,559 (~95.1%) genes were clustered into 544,415 orthogroups in seven various plants (Table S2). Among these orthogroups, we identified 21,651 common orthogroups, including 70,412 genes, which was the greatest number in clustered orthogroups (Figure 3a; Tables S2 and S3). Furthermore, a phylogenetic tree was constructed for the seven *Brassicaceae* genomes using 10,950 single-copy orthologs genes, with *B. rapa* as an outgroup. As expected, Korso and HDEM, with a common curd trait, were clustered into one clade. OX-heart, D134, and line02-12, with a heading leaf trait, were clustered into another clade (Figure 3b). Two historical events can be observed clearly through synonymous substitution rate (Ks) analysis: a whole genome triplication (WGT) of *Brassica* species is estimated to have occurred approximately 11 million years ago (Mya) (Figure 3b,c; Table S4); the differentiation time between *B. rapa* and *B. oleracea* is ~2.85 to 3.04 Mya (Figure 3b,c; Table S4), displaying a divergence time between the AA and CC genomes of diploid *Brassica* plants.

### 2.3. Genomic Variation of Six B. oleracea Genomes

**Figure 4:**
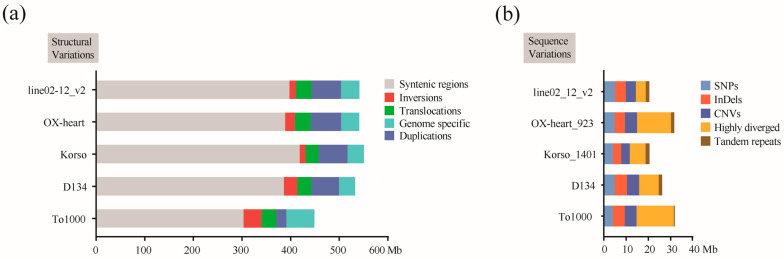
Structural and sequence variations between seven *B. oleracea* genomes. The bar plots show the total span of syntenic and structural variational (**a**) and sequence variational (**b**) regions between the HDEM and each of the other accessions.

The genetic variation, including sequence and structural variations, among six *B. oleracea* genomes using HDEM as a reference genome were detected. Approximately 302.9–418.3 Mb (22,627–35,459) syntenic blocks (Figures S2 and S3a) of other accessions were anchored in the HDEM genome (Figure 4a; Table S5). In this block, we identified 20.5–31.7 Mb (5,119,342–6,524,666) sequence differences (Figure S3b), which included 4.04–4.99 × 10^6^ SNPs and 3.79–5.49 × 10^6^ InDels in each of the genomes (Figure 4b; Table S5). The InDels were most abundantly less than 500 bp (Figure S4). Then, we annotated the SNPs and InDels based on the annotation file of HDEM (Table S6). The SNP and InDels causing start loss, stop gain, stop loss, and frameshift of genes were regarded as large-effect variations. Notably, large-effect variations in Korso were less than in others using HDEM as a reference genome (Figure S5). This suggested that the cauliflower morphotype Korso is similar to the broccoli morphotype HDEM at the molecular level.

We also determined 132.3–152.5 Mb (95,167–177,059) SV regions among HDEM and other lines (Figure 4a and Figure S3; Table S5). Remarkably, we found that the accession of Korso belonging to cauliflower had the most syntenic regions and the least structural regions compared with the others. This implied that there is a low degree of differentiation between cauliflower and broccoli. In the SV regions, chromosome rearrangement events containing 12.3–36.6 Mb (175–229) inversions, 26.7–32.4 Mb (7354–15,382) translocations, and 21.1–62.7 Mb (19,470–24,326) duplications were recognized within each of the individual genomes (Figure 4a; Table S5). The lengths of inversions, translocations, and duplications ranged from 1 to 100 k (Figure S6). For instance, we detected a 58 kb inversion between Korso and OX-heart (Figure S7), which may be associated with complex domestication traits. These results highlighted the extensive SVs during the intraspecific diversification of the *B. oleracea* genome. 

### 2.4. Structural Variations within Genes Associated with the Heading Trait of Cabbage

**Figure 5:**
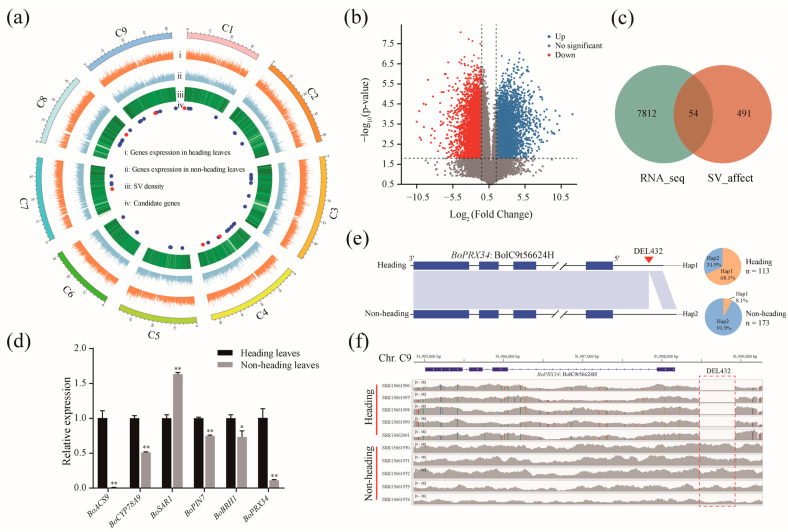
SVs in genes involved in heading leaf trait of cabbage. (**a**) Circular diagrams of gene expression and genomic variation between heading and non-heading accessions. The outermost layer illustrates the distribution of the nine chromosomes of *B. oleracea* HDEM in megabases (Mb). Tracks of (ⅰ) and (ⅱ) show the distribution of expression of genes from heading and non-heading leaves with three biological replicates, respectively. Gene expression was calculated as the fragments per kilobase of transcript per million mapped reads (FPKM) and normalized by log_10_(FPKM + 1). (ⅲ) The density of SVs in 200 kb bins. (ⅳ) Location of candidate genes, including six identified genes highlighted by the red solid circle for heading trait. (**b**) Volcano plot showing the fold change (x-axis) and significance level of the differential expression (y-axis) between heading leaves and non-heading leaves of cabbage, based on normalized RNA-seq read counts. The gray dashed lines represent the threshold for determining differentially expressed genes, and those indicating differential expression are colored red and blue. (**c**) A Venn diagram showing the overlapping genes expressed significantly at different levels and affected by SVs. (**d**) Relative expression levels of *BoACS9*, *BoCYP78A9*, *BoSAR1*, *BoPIN7*, *BoBRH1*, and *BoPRX34* between heading and non-heading leaves of the cabbage. The expression level in heading leaves was set as 1.0. Each value represents the mean ± SD (n = 3 replicates). Student’s *t*-test analysis was used to determine significant differences between heading and non-heading leaves. **, *p* ˂ 0.01 and *, *p* ˂ 0.05. (**e**) (**left**) Local collinearity analysis between the two haplotypes of the *BoPRX34* gene. Hap, haplotype. (**right**) Comparison of the two haplotypes of the *BoPRX34* between heading and non-heading populations. (**f**) Short reads of different accessions were mapped to the *BoPRX34* gene at 31,905–31,909 kb of chromosome C9 of HDEM. The red dotted rectangle shows the location of DEL432.

To identify the SVs responsible for the heading trait of cabbage, we downloaded short read data of 288 *B. oleracea* accessions, including 113 cabbage accessions with the heading trait and 173 other morphotype accessions with the non-heading trait (Table S7). Then, we carried out SV calling among 288 *B. oleracea* accessions using HDEM as the reference genome. We found 1205 SVs that might be related to the heading trait (Figure 5a) because the allele of these SVs was enriched in the cabbage morphotype. A total of 545 potential candidate genes near SVs were identified. In addition, we analyzed RNA-seq data from three biological replicates of heading and non-heading leaves of a cabbage accession named OX-heart (Figure 5b). A total of 7965 differentially expressed genes were identified between heading leaves and non-heading leaves (Figure 5b,c). By overlapping two gene sets of SV-affected and RNA-seq, we obtained 54 candidate genes that may be involved in heading trait formation (Figure 5c; Table S8). Fifty orthologous genes were detected in the *Arabidopsis thaliana* genome (Table S8). With the aid of gene function annotations of the *A. thaliana* genome and the tair website (TAIR—Home Page (arabidopsis.org)), we found that the *BoPRX34*, *BoACS9*, *BoCYP78A9*, *BoSAR1*, *BoPIN7*, and *BoBRH1* genes are involved in cell elongation and phytohormones, which may play a crucial role in heading formation of *B. oleracea* (Table S9). Given the fact that the heading morphotype is a domestication trait, we performed selection analyses between cabbage (n = 113) and other morphology (n = 173) groups using pairwise fixation statistics (*F*_ST_) values for six genes. We found that six genes were subjected to selection (Figure S8). Additionally, the result of qRT-PCR experiments shows the relative expression level of six genes was significantly different between heading leaves and non-heading leaves (Figure 5d). For the *BoPRX34* gene, with homology to the *PRX* gene located in the cell wall and implicated in cell elongation in *Arabidopsis* [24], there is a 432 bp deletion downstream of *BoPRX34* (Figure 5e,f). This haplotype accounted for 68.1% of the heading accessions but only 8.1% of the non-heading accessions (Figure 5e), suggesting that the 432 bp deletion was associated with the heading trait. In parallel, we identified a 945 bp duplication, 974 bp deletion, 742 kb insertion, 1941 bp deletion, and 1101 bp insertion in the *BoACS9*, *BoCYP78A9*, *BoSAR1*, *BoPIN7*, and *BoBRH1* genes, respectively (Figure S9). The allele frequencies of these five variations varied significantly between the heading and non-heading populations (Figure S8). These results mean that the six candidate genes accompanied by SVs may be responsible for the heading trait of cabbage.

## 3. Discussion

With the assistance of new sequencing technology, several high-quality *B. oleracea* reference genomes have been released [1,15,16,18,19,25]. The reference genomes facilitated the study of the genome and pangenome of *B. oleracea* plants. For example, on the basis of the line02-12 genome, Cheng et al. [4] investigated the evolutionary relationships of *B. oleracea* by resequencing 119 different morphotypes of *B. oleracea* and identified important candidate genes involved in the leaf heading trait of cabbage and stem tuberous of kohlrabi. Similarly, using the To1000 genome, researchers performed RNA-seq sequencing of 224 various types of *B. oleracea* to explore the domestication and evolutionary history of *B. oleracea* [21]. In addition, with To1000 as a reference genome, Golicz et al. [26] constructed the first *B. oleracea* pangenome and elucidated that genes affected by presence/absence and copy number variation contributed to phenotypic diversity. Despite the proliferation of population genomics studies based on these genomes, a comprehensive comparative genomics analysis is lacking. Hence, we used chromosome-scale broccoli named HDEM as a reference genome, and other *B. oleracea* genomes were aligned to it to obtain genomic variation, including sequencing and structural variations. A total of 132.3–152.5 Mb (95,167–177,059) SVs were identified between six *B. oleracea* genomes, more than those in seven *A. thaliana* genomes, with only 12.6–17.0 Mb [27]. Considering the abundant diversity of *B. oleracea*, the vast SVs between *B. oleracea* genomes may be associated with morphotype domestication, similar to the SVs tracking *B. rapa* morphotype domestication [28]. Interestingly, we found 132.3 Mb (95,167) SVs between the HDEM and Korso genomes, which were less than those between HDEM and other morphotype genomes, and 418.3 Mb syntenic regions between the HDEM and Korso genomes, which were more than those in the other genomes. This implied that broccoli resembles cauliflower not in morphotype but at the molecular level. A phylogenetic relationship tree showed that cauliflower and broccoli were clustered into the same clade, meaning that they were less differentiated from each other, which supported the viewpoint that broccoli and cauliflower probably originated from the same place [2]. 

SVs have been reported to be responsible for many important agronomic traits, such as grain size in rice [29], flowering time in wheat [30], fruit shape in tomato [31], and plant architecture in cotton [32]. The heading leaf of cabbage is a typical trait for understanding the power of artificial selection in *B. oleracea*. Three candidate genes, *BoKAN2.2*, *BoBRX.2*, and *BoATHB15.2*, involved in heading leaf formation of *B. oleracea,* have been reported by exploring SNPs [4]. However, there are few studies of heading traits using SVs in *B. oleracea*. In this study, we identified six candidate genes by integrating SV calling in 288 *B. oleracea* accessions and transcriptome analysis between heading and non-heading leaves. By paring the genetic basis of these six genes, we found that they were all affected by SV, which caused changes in gene expression. Among them, we found that the *BoPRX34* gene is orthologous to *PRX34* in *Arabidopsis*. The *PRX34* gene is a dominant-negative regulator of plant growth and development. It is located in the cell wall and involved in cell elongation [33,34]. Additionally, the *PRX34* mutation causes larger leaves than wild-type leaves and a delayed senescence phenotype in *Arabidopsis* [24]. In addition, we discovered that the other five candidate genes affected by SVs were involved in hormone signaling. For example, *ACS9* is an essential gene that acts within the ethylene biosynthetic process [35]. Its mutant increased mature plant height, reduced branching, reduced the number of rosette leaves, and decreased ethylene production in *Arabidopsis* [36]. The *CYP78A9* gene encoding a cytochrome P450 monooxygenase is a dominant suppressor of brassinosteroid-responsive gene expression. It is involved in *Arabidopsis* reproductive development [37]. Overexpression of the *CYP78A9* gene induces large and seedless fruit in *Arabidopsis* [38]. The *suppressor of auxin resistance1* (*SAR1*) is a suppressor of the auxin resistance gene. Additionally, the loss of SAR1 protein results in a severe growth phenotype [39]. *PIN7* is a modulator of auxin transport, resulting in a decrease in auxin transport from the root to the shoot [40,41]. Additionally, the *brassinosteroid-responsive RING-H2* (*BRH1*) is a dominant gene involved in brassinosteroid-mediated signaling pathway and altered leaf shapes in *Arabidopsis* [42,43]. These results provide novel insight into the *B. oleracea* plant development.

## 4. Materials and Methods

### 4.1. Long Terminal Repeat (LTR) Assembly Index (LAI) Calculation

To evaluate the quality of the six *B. oleracea* genomes, we calculated the LAI in HDEM [16], line02-12_v2 [18], OX-heart_923 [19], Korso_1401 [19], D134 [25], To1000 [15], and line02-12_v1 [1]. Briefly, the LTR retrotransposons of six genomes were identified using LTR_FINDER (v1.07) [44] with the following parameters: -D 15,000 -d 1000 -L 7000 -l 100 -p 20 -C -m 0.9 and LTRharvest (v1.6.1) [45] with the parameters setting: -similar 85 -vic 10 -seed 20 -seqids yes -minlenltr 100 -maxlenltr 7000 -mintsd 4 -maxtsd 6 -motif TGCA -motifmis 1. Subsequently, high-confidence full-length LTR retrotransposons (LTR-RTs) were extracted by LTR_retriever (v2.8) software [46] using the output file from LTR_FINDER [44] and LTRharvest [45]. Then, the final file “*.out.LAI ” provided detailed information on the LAI of the whole genome and each chromosome.

### 4.2. Synteny and Ks Analysis

To identify syntenic blocks, the CDSs from *B. rapa* Chiifu_v3 [47] and *B. oleracea* encompassing HDEM [16], OX-heart_923 [19], Korso_1401 [19], line02-12_v2 [18], D134 [25], and To1000 [15] were used to conduct all-against-all LAST (v1021) [48] between inter- and intraspecies. Based on the LAST [48] results, syntenic regions were discerned using JCVI (v0.9.14) [49]. The dot plots between *B. oleracea* and *B. rapa* were also drawn by JCVI [49]. The synonymous substitution rates (Ks) values with the NG86 model of homologous gene pairs within each collinear block were calculated by WGDI [50], a useful toolkit for evolutionary analyses. Then, Ks values of all pairwise genes were plotted by the ggplot2 package (v3.2.0) [51] in R software (https://www.r-project.org/, accessed on 26 June 2022, version 3.6.1). Generally, the peak of intraspecific Ks corresponds to genome duplication events, and the peak of interspecies Ks corresponds to divergence events. *Brassica* plants were reported to have experienced a specific whole genome triplication (WGT) event [1,12,52], and the value of synonymous replacement rate (r) is 1.5 × 10^−8^ mutations site/year in *Brassica* plants described in previous research [53]. Subsequently, the time of WGT and differentiation was estimated using the formula T = Ks peak/2r.

### 4.3. Gene Family Cluster and Phylogenetic Analysis of B. oleracea

We used the protein-coding genes Chiifu_v3 [47], Korso_1401 [19], OX-heart_923 [19], HDEM [16], D134 [25], line02-12_v2 [18], and To1000 [15] from the *Brassica* database (BRAD V3.0) [54] to construct orthogroups by OrthoFinder [23]. In detail, the longest isoform of each gene among the seven species was extracted by the Fasta Get Representative procedure of TBtools (v1.09876), which was further compared using the blast fast tool Diamond (v0.8.22.84) [55]. Then, the Markov cluster algorithm (MCL) [56] was used to cluster genes into orthogroups. From the clustering results, the protein sequences of single-copy genes were aligned using MAFFT (v7.475) [57]. Subsequently, the multiple sequence alignment was optimized by Gblocks (v0.91b) [58]. The phylogenetic tree was constructed using RAxML (v8.2.12) [59] with the following parameters “-f a -x 12345 -p 12345 -N 1000 -m PROTGAMMAAUTO -T 20”. The tree was drawn using the user-friendly tool iTOL [60] (https://itol.embl.de/, accessed on 21 June 2022).

### 4.4. Genomic Variations Identification

All five *Brassica oleracea* (CC) genomes, containing OX-heart_923 [19], Korso_1401 [19], line02-12_v2 [18], To1000 [15], and D134 [25], were aligned to the HDEM [16] genome using MUMmer (v4.0) [61] with the parameters: --mum -c 100 -b 500 -l 50 -t 10. Then, the results of the raw alignments were filtered using a “delta-filter” with the following parameters: -m -i 90 -l 100. Furthermore, the “show-coords” function with the “-THrd” setting was used to transform the filter to standard files with coordinates for the next input. Then, we used Synteny and Rearrangement Identifier (SyRI) (v1.4) [62], with default parameters, to detect genomic variations. The genomic variations from SyRI [62] comprise sequence differences and structural differences. The sequence differences included SNPs, InDels, CNVs, highly diverged, and tandem repeats, and the SNPs and InDels were used for further analysis. The structural differences contain inversions, translocations, duplications, and genome-specific differences.

### 4.5. Genomic Variation Annotation

First, we downloaded the annotated file and genomic sequences of HDEM [16] (https://www.genoscope.cns.fr/externe/plants/chromosomes.html, accessed on 31 October 2021), and then we fixed the annotated file using the “GXF Fix” function of TBtools (v1.09876) [63] for ANNOVAR software (latest version) [64]. Three steps were used to build a database of HDEM: (1) transforming the gff file to gtf file using gffread (v0.11.6) [65]; (2) converting the gtf file to genePred file using gtfToGenePred (http://hgdownload.cse.ucsc.edu/admin/exe/linux.x86_64/gtfToGenePred, accessed on 29 June 2022); (3) constructing the refGene using a retrieve_seq_from_fasta.pl script of ANNOVAR [64]. Finally, the VCF files generated by SyRI (v1.4) software [62] were annotated directly by two scripts of ANNOVAR [64]: convert2annovar.pl and table_annovar.pl.

### 4.6. Identification of SVs Involved in Heading Trait

To identify high-quality SVs, we mapped the Illumina reads of 288 different morphotype *B. oleracea* accessions to the reference HDEM genome using BWA (v0.7.12-r1039) [66] with the following parameters: bwa mem. Then, two software programs, smoove (v0.2.8) (https://github.com/brentp/smoove, accessed on 19 August 2022) and DELLY (v1.1.3) [67], were used to perform SV calling. To improve the accuracy, the SVs detected by both tools were retained. Finally, we merged and filtered the SVs using SURVIVOR (v1.0.7) [68] with the following parameters: SURVIVOR merge 1000 2 1 1 0 50. We characterized the SVs related to the heading trait by comparing the allele frequency in cabbage and other morphotype populations. The SVs with allele frequencies in the heading population that were 2.5 or 0.4 times that in the non-heading populations were considered putative SVs that may be related to the heading trait of cabbage. The *F*_ST_ values were calculated manually between cabbage and others morphotype groups.

### 4.7. RNA-Seq Analysis

The raw RNA-seq data of heading and non-heading leaves from a cabbage accession named OX-heart [19] were downloaded from the National Center for Biotechnology Information (NCBI) (https://www.ncbi.nlm.nih.gov/, accessed on 10 April 2022). The data were used to predict and annotate the protein-coding genes of the OX-heart genome in a previous study [19]. Three biological repetitions of non-heading leaves included 7.0 Gb of raw data, and the numbers of SRRs are SRR13759385, SRR13759402, and SRR13759403. Three biological repetitions of heading leaves included 6.2 Gb raw data, and the numbers of SRRs are SRR13759388, SRR13759387, and SRR13759386. The clean data filtered from two types of leaves were mapped to the *B. oleracea* HDEM reference genome with hisat2 (v2.2.1) [69] using the default parameters. Then, the mapped reads in SAM format were converted into BAM format and sorted using SAMtools (v1.4.1) [70]. The read count value was calculated by htseq-count (v0.13.5), a subfunction of HTSeq (v2.0) [71]. The gene-level relative abundances in several fragments per kilobase per million (FPKM) values were determined by a Perl script. Differentially expressed genes between the two groups were identified using DESeq [72] based on *p*  ≤  0.05 and |log_2_ Fold Change|  ≥  1.

### 4.8. RNA Extraction and Quantitative Real-Time (qRT)-PCR

Total RNA from heading and non-heading leaves of *B. oleracea* var. *capitata* (cabbage) were isolated using TransZol (TRAN, Beijing, China). Reverse transcription was performed using a HiScript II Q RT SuperMix for qPCR (+gDNA wiper) kit (Vazyme, Nanjing, China). qRT-PCR was performed using LightCycler 480 High-Resolution Melting Master Mix (Roche, Switzerland) following the manufacturers’ instructions on a Roche LC 480 machine. Relative gene expression levels were calculated using the 2^−∆∆Ct^ method [73]. The primer pairs used in the qPCR experiment are shown in Supplementary Table S10. 

## 5. Conclusions

In this study, we conducted a comprehensive comparative genetic analysis among six *B. oleracea* and identified extensive SVs by comparative population genomics. By analyzing these valuable SVs, we identified six important genes involved in the heading trait of cabbage. The RNA-seq and qRT-PCR experiments give further validation of six identified genes. These six genes could be used for developing molecular markers linked to heading traits for further marker-assisted selection (MAS) in *Brassica* crops.

## Data Availability

Not applicable.

## Supplementary Materials

The following supporting information can be downloaded at: https://www.mdpi.com/article/10.3390/ijms24044063/s1.
