# Supplementary material for: Whole-Genome Comparison Reveals Structural Variations behind Heading Leaf Trait in Brassica oleracea
Source: Int J Mol Sci. 2023 Feb 17;24(4):4063. doi: 10.3390/ijms24044063 (PMC9965001; doi:10.3390/ijms24044063)
Supplement: Supplementary file 1 [file ijms-24-04063-s001.zip › Supplementary FiguresS1-9.pdf]

Supplementary Figures S1-S9:

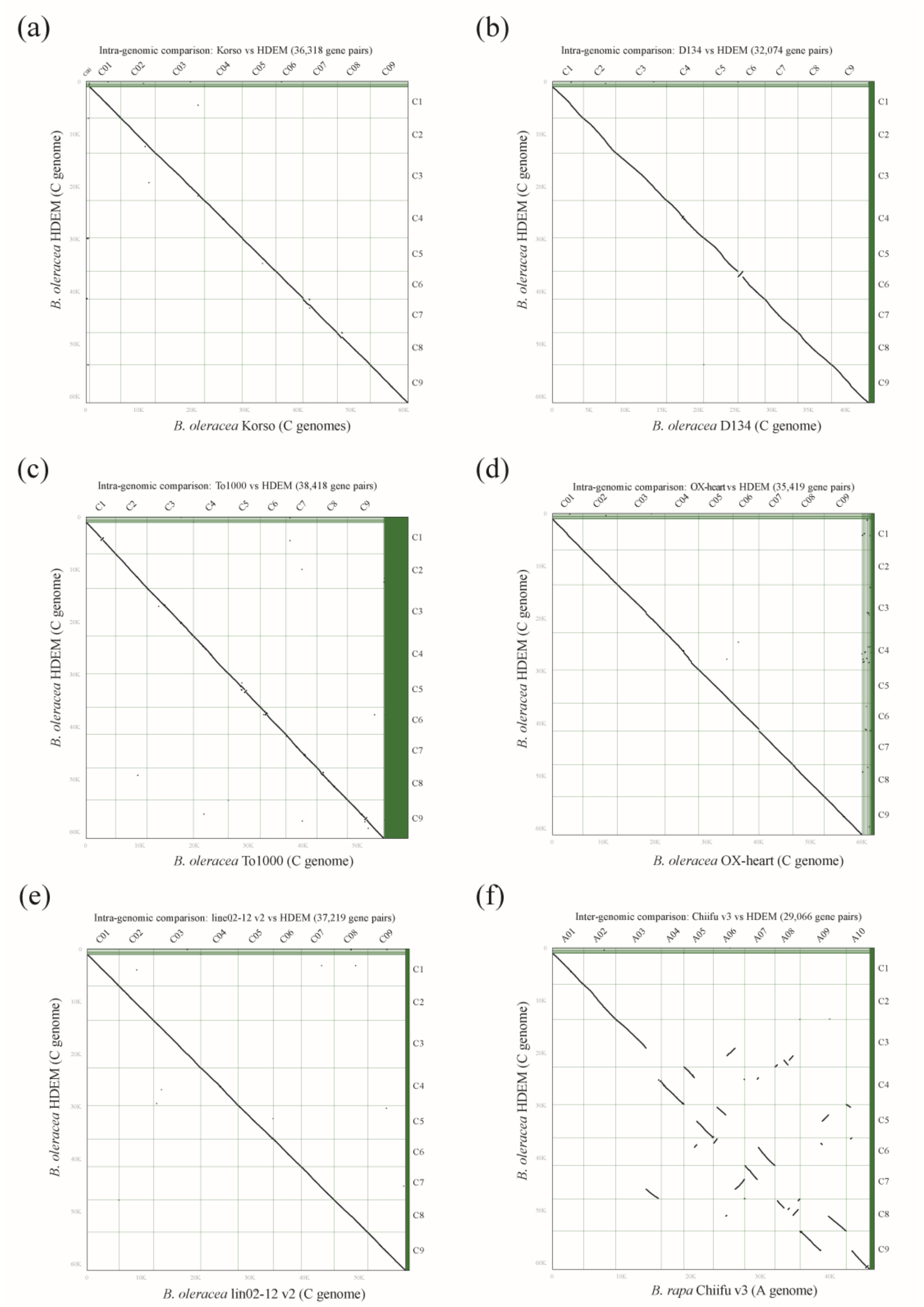

Supplementary Figure S1. Genomic synteny block exhibition by dotplot. Whole-genome synteny among *B. oleracea* HDEM [1] (y-axis) and B. oleracea Korso [2] (a), D134 [3] (b), To1000 [4] (c), OX-heart [2] (d), line02-12_v2 [5] (e), and *B. rapa* Chiifu v3 [6] (f) (x-axis) were depicted with dots after 1:1 synteny screen. Axes are scaled by gene numbers.

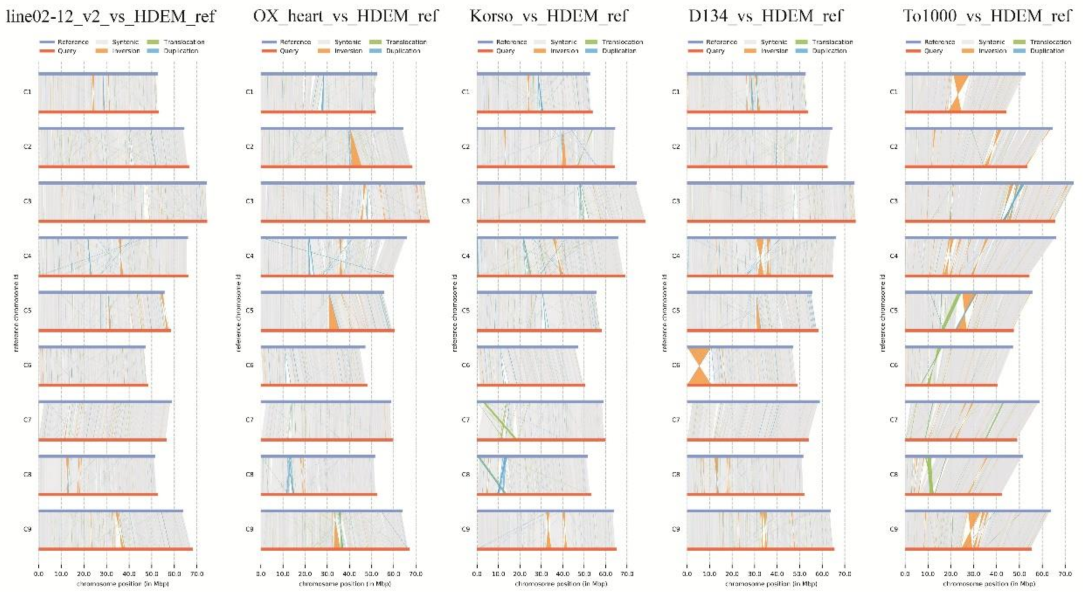

Supplementary Figure S2. The overview of genome-wide comparison between *B. oleracea* genomes. The left of the panel each graph indicated the chromosome ID of the HDEM genome. Silvery, yellow, green, and light blue lines represent the syntenic regions, inversions, intra-translocations, and duplications, respectively. The plot was drawn by SyRI (v1.4) [7] tools.

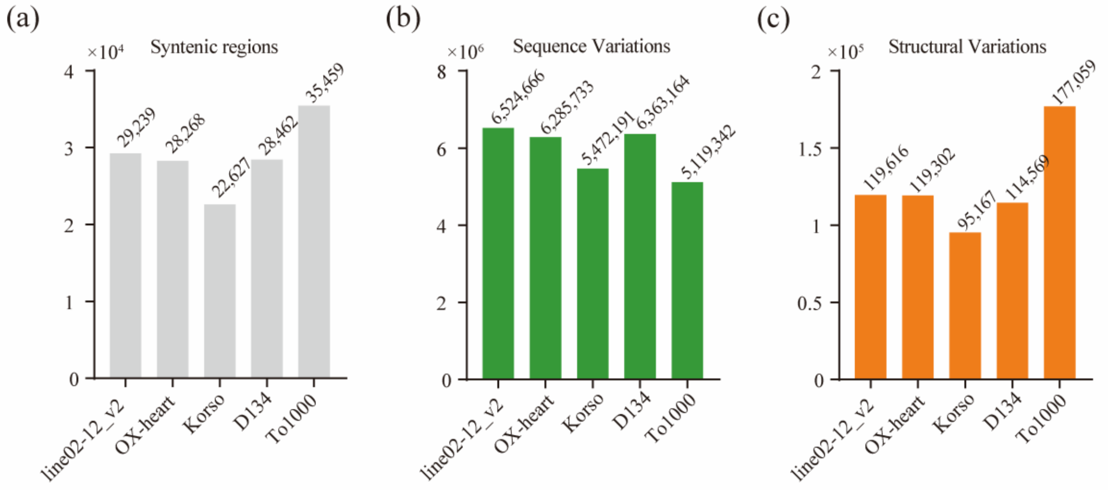

Supplementary Figure S3. The Statistics of genomic variation among six *B. oleracea* genomes. The bar plots show the number of syntenic blocks (a), sequence variations (b) and structural variations (c).

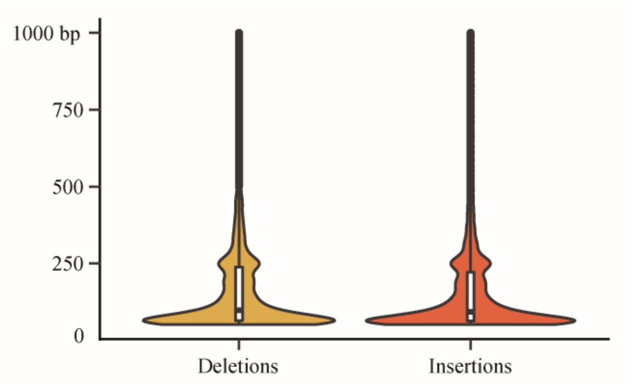

Supplementary Figure S4. Size distributions of deletions and insertions.

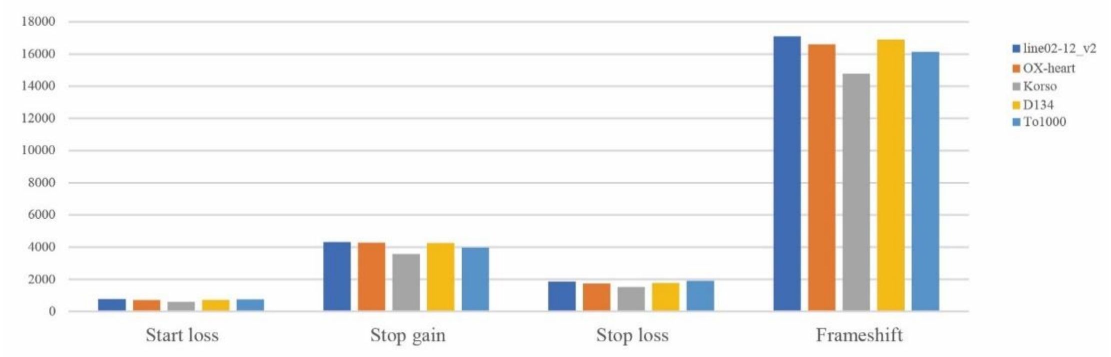

Supplementary Figure S5. The number of big-effect variations in various *B. oleracea* genomes.

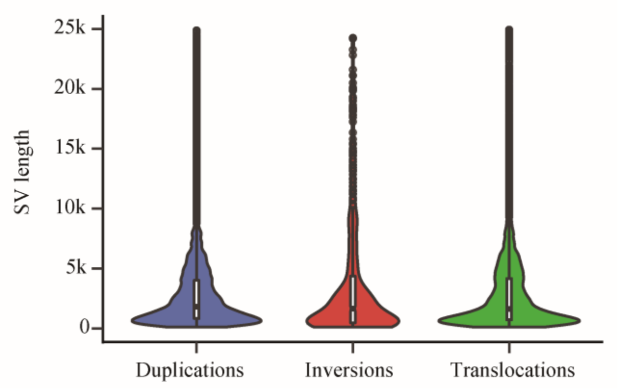

Supplementary Figure S6. Size distributions of duplications, inversions, and translocations.

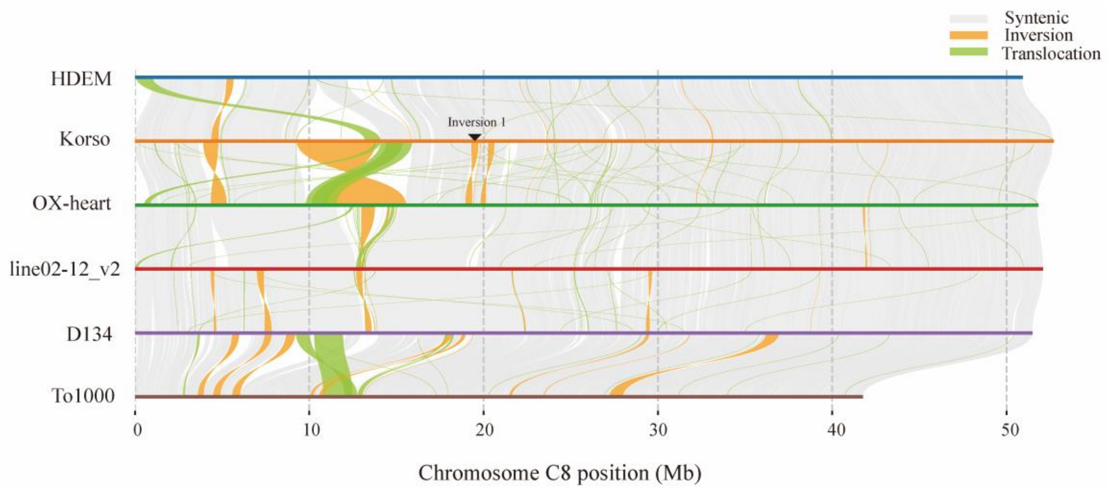

Supplementary Figure S7. Schematic of the structural variations on chromosome C8 among six genomes.

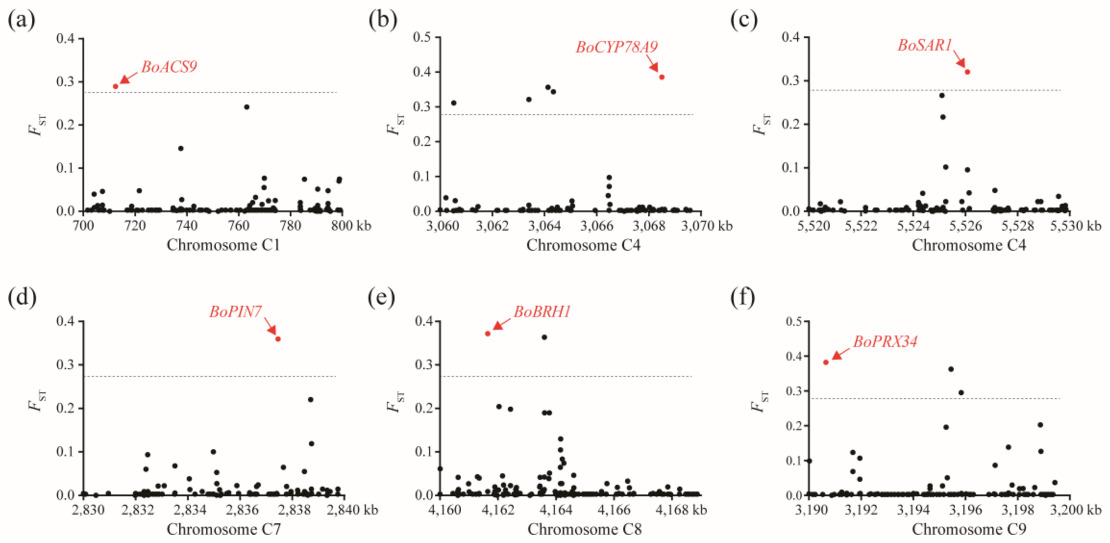

Supplementary Figure S8. The six identified genes are associated with *B. oleracea* heading morphotype domestication. (a) The F_{ST} values represented by dot plots were calculated from 700 to 800 kb on chromosome C1. The horizontal dashed line presents the empirical threshold of F_{ST} = 0.28. The arrow indicates the location of the *BoACS9* gene affected by the SV. The selective signals of *BoCYP78A9*, *BoSAR1*, *BoPIN7*, *BoBRH1*, and *BoPRX34* genes are shown in (b), (c), (d), (e), and (f), respectively.

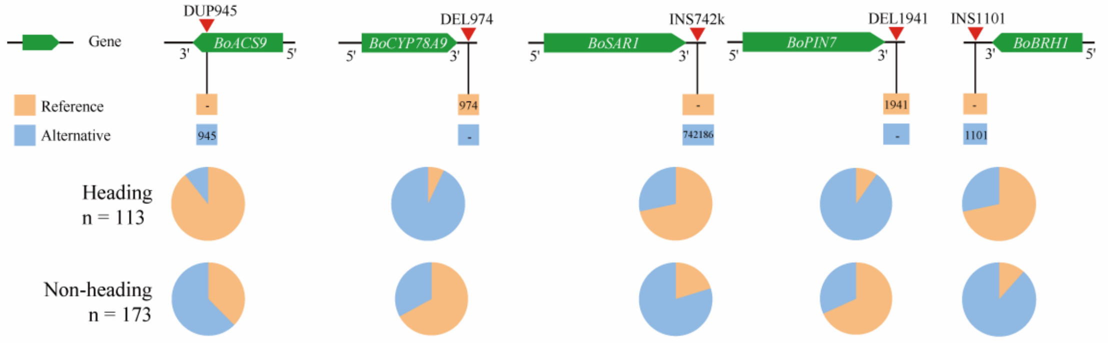

Supplementary Figure S9. (top) The genotype of five candidate genes affected by SVs. (bottom) the pies show the percentage of the SVs within candidate genes in heading and non-heading populations, respectively. "n" indicates the number of individuals per population.

Reference

1. Belser, C.; Istace, B.; Denis, E.; Dubarry, M.; Baurens, F.-C.; Falentin, C.; Genete, M.; Berrabah, W.; Chèvre, A.-M.; Delourme, R.; et al. Chromosome-scale assemblies of plant genomes using nanopore long reads and optical maps. *Nat. Plants* **2018**, *4*, 879–887.
2. Guo, N.; Wang, S.; Gao, L.; Liu, Y.; Wang, X.; Lai, E.; Duan, M.; Wang, G.; Li, J.; Yang, M.; et al. Genome sequencing sheds light on the contribution of structural variants to *Brassica oleracea* diversification. *BMC Biol.* **2021**, *19*, 93.
3. Lv, H.; Wang, Y.; Han, F.; Ji, J.; Fang, Z.; Zhuang, M.; Li, Z.; Zhang, Y.; Yang, L. A high-quality reference genome for cabbage obtained with SMRT reveals novel genomic features and evolutionary characteristics. *Sci. Rep.* **2020**, *10*, 12394.

4. Parkin, I.A.P.; Koh, C.; Tang, H.; Robinson, S.J.; Kagale, S.; Clarke, W.E.; Town, C.D.; Nixon, J.; Krishnakumar, V.; Bidwell, S.L.; et al. Transcriptome and methylome profiling reveals relics of genome dominance in the mesopolyploid *Brassica oleracea*. *Genome Biol.* **2014**, *15*, R77.
5. Cai, X.; Wu, J.; Liang, J.; Lin, R.; Zhang, K.; Cheng, F.; Wang, X. Improved *Brassica oleracea* JZS assembly reveals significant changing of LTR-RT dynamics in different morphotypes. *Theor. Appl. Genet.* **2020**, *133*, 3187–3199.
6. Zhang, L.; Cai, X.; Wu, J.; Liu, M.; Grob, S.; Cheng, F.; Liang, J.; Cai, C.; Liu, Z.; Liu, B.; et al. Improved *Brassica rapa* reference genome by single-molecule sequencing and chromosome conformation capture technologies. *Hortic. Res.* **2018**, *5*, 50.
7. Goel, M.; Sun, H.; Jiao, W.; Schneeberger, K. SyRI: Finding genomic rearrangements and local sequence differences from whole-genome assemblies. *Genome Biol.* **2019**, *20*, 277.
